# Volume of Amygdala Subregions and Plasma Levels of Brain-Derived Neurotrophic Factor and Cortisol in Patients with s/s Genotype of Serotonin Transporter Gene Polymorphism of First-Episode and Drug-Naive Major Depressive Disorder: An Exploratory Study

**DOI:** 10.3390/neurolint14020031

**Published:** 2022-04-15

**Authors:** Naomichi Okamoto, Keita Watanabe, Hirofumi Tesen, Atsuko Ikenouchi, Ryohei Igata, Yuki Konishi, Tomoya Natsuyama, Rintaro Fujii, Shingo Kakeda, Taro Kishi, Nakao Iwata, Reiji Yoshimura

**Affiliations:** 1 Department of Psychiatry, Hospital of University of Occupational and Environmental Health, Kitakyushu 807-8555, Japan; nokamoto@med.uoeh-u.ac.jp (N.O.); h-tesen@med.uoeh-u.ac.jp (H.T.); atsuko-i@med.uoeh-u.ac.jp (A.I.); igataryouhei@gmail.com (R.I.); konikoni0125@outlook.jp (Y.K.); natsuyama@med.uoeh-u.ac.jp (T.N.); rintarou_5@hotmail.com (R.F.); 2 Medical Center for Dementia, Hospital of University of Occupational and Environmental Health, Kitakyushu 807-8555, Japan; 3 Open Innovation Institute, Kyoto University, Kyoto 606-8501, Japan; sapient@med.uoeh-u.ac.jp; 4 Department of Radiology, Graduate School of Medicine, Hirosaki University, Hirosaki 036-8560, Japan; kakeda@med.uoeh-u.ac.jp; 5 Department of Psychiatry, Fujita Health University, Toyoake 470-1192, Japan; tarok@fujita-hu.ac.jp (T.K.); nakao@fujita-hu.ac.jp (N.I.)

**Keywords:** amygdala, subregions, major depressive disorder, brain-derived neurotrophic factor, cortisol, serotonin transporter gene polymorphism

## Abstract

The amygdala is a prominent region of the brain that plays a critical role in the pathophysiology of major depressive disorder (MDD). The amygdala is formed from a collection of interconnected substructures (nuclei) that relay signals from multiple brain areas, which suggests that the amygdala has different functions depending on its subregion. There are two main alleles of serotonin transporter gene polymorphism (*5-HTTLPR*): a 44-bp insertion (l-allele) or deletion (s-allele). The transcriptional activity of the l-allele of the gene is twice that of the s-allele. The present study aimed to investigate the association between the volume of the whole amygdala and subregions of the amygdala in 25 first-episode and drug-naive patients with MDD and 46 healthy controls (HCs) with the s/s genotype of *5-HTTLPR* and plasma levels of brain-derived neurotrophic factor (BDNF) or cortisol. No significant difference was observed in the amygdala total and subregion volumes between the HC and MDD groups. No significant difference was found in the plasma levels of BDNF and cortisol between the two groups. In addition, no correlations were found between the total and subregion amygdala volume and plasma levels of cortisol or BDNF.

## 1. Introduction

Major depressive disorder (MDD) is accompanied by morphological changes in the brain structure, which are of great importance in the neural circuitry mediating the hippocampus and amygdala [1]. The amygdala, the emotional center of the brain, is particularly involved in feelings of anxiety and fear and has been linked to several mental disorders. For example, in patients with MDD, the activity and volume of the amygdala are known to be different from those of healthy people [2,3]. Recent brain imaging studies using magnetic resonance imaging (MRI) have demonstrated several interesting findings. For example, on the one hand, some studies of the amygdala have reported reduced volume due to stressful life events [3,4]. On the other hand, some reports have suggested enlargement of the amygdala in patients with MDD, which illustrates conflicting findings and inconsistency in the current knowledge in this field [5]. Moreover, the lateral nucleus of the amygdala is its main input structure and is required for all aspects of threat learning and associative plasticity [6]. The structural changes in the lateral nucleus of the amygdala and the anterior amygdaloid area could be related to changes in neural circuitry, which possibly result from the development and progression of diseases. The limbic-cortico-striatal-pallidal-thalamic (LCSPT) circuit plays an important role in understanding the neuroanatomical pathophysiology of depression [7,8,9]. Rather than being a unitary structure, the amygdala is formed from a collection of interconnected substructures (nuclei) that relay signals from multiple brain areas [5]. Different regions of the amygdala have different functions [10,11]. Functional neuroimaging studies have provided strong support for the critical role the amygdala plays in emotional processing [12]. It was suggested that a potential mechanism of sensitization to intense stress and depression may represent a reduction in hippocampal and amygdala volume [4].

Serotonin transporter gene polymorphism (*5-HTTLPR*) is one of the major genetic factors determining inter-individual differences in stress reactivity. Serotonin transporter (5-HTT) mediates the reuptake and recycling of the released serotonin following neuronal stimulation [13]. There are two main alleles: a 44-bp insertion (l-allele) or a deletion (s-allele). The transcriptional activity of the l-allele of the gene is twice that of the s-allele [14]. Therefore, s-allelic variants have less 5-HT reuptake than the l-allelic variants because of the reduced expression of 5-HTT mRNA [15,16]. This could mean that individuals with the s/s genotype are more sensitive to all stressful life events than those with the s/l or l/l genotype. It has also been shown that 5-HTT knockout mutations moderate the adaptive response to early adverse environmental factors [17,18]. The latter study used heterozygous 5-HTT knockout mice (having a 50% gene dose-dependent reduction of 5-HTT expression) and found that, although these mice did not show behavioral deficits when raised by mothers providing a lot of maternal care, they developed increased anxiety and depression-related behavior in adulthood when raised by mothers providing poor maternal care. It was proposed that such a gene-by-environment interaction could serve as a model for the increased vulnerability to early life stress in individuals with the *5-HTTLPR* s-allele [19].

It is speculated that connections from the amygdala to the hypothalamus activate the hypothalamic-pituitary-adrenal (HPA) axis and cortisol response [5]. In addition, it has been reported that individuals with the s/s genotype show enhanced cortisol secretion in response to acute psychological stressors [20], which may increase cortisol levels, and may be associated with alterations in the prefrontal network in the early stage of MDD [21]. Brain-derived neurotrophic factor (BDNF) is a neurotrophin that is vital for the survival, growth, and maintenance of neurons in key brain circuits involved in emotional and cognitive functions. For instance, BDNF is associated with neuroplasticity in the brain and plays a role in the pathophysiology of MDD [22]. A previous study suggested that plasma/serum levels of BDNF were decreased in patients with acute MDD compared to healthy controls (HCs) [23,24]. Several studies have shown that monoaminergic neurotransmission involving 5-HT, norepinephrine, and dopamine exerts a major influence on the brain circuits involved in the regulation of mood, reactivity to psychological stress, self-control, motivation, drive, and cognitive performance in a complicated manner [25]. BDNF and cortisol have distinct roles in the physiology of the brain, but at the same time, there is an interaction between them [26]. It is plausible that BDNF has an influence on the cortisol responsivity to stress, thereby implying a Met-allele in *Val66Met* polymorphism and cortisol integrative system. BDNF and cortisol undoubtedly play distinct and complementary roles in the physiology of the nervous system, in which cortisol proves to be the regulator of positive and negative effects [27,28]. However, the detailed mechanism of the interaction between BDNF and cortisol for the pathophysiology of MDD remains unknown.

Since the relationship between the amygdala subregion volume and the plasma levels of BDNF and cortisol have not been sufficiently studied, we aimed to elucidate this point in first-episode and drug-naive MDD patients with the s/s genotype of *5-HTTLPR*. Recent developments in image analysis techniques have made it possible to measure the volumes of the subregions of the amygdala in greater detail. The present study aimed to investigate the correlation between the volume of the whole amygdala and subregions of the amygdala in 25 first-episode and drug-naive MDD patients and 46 HCs with the s/s genotype of *5-HTTLPR*, and plasma levels of BDNF and cortisol. To the best of our knowledge, this is the first study focusing on the volume of amygdala subregions and peripheral levels of BDNF or cortisol in MDD patients with the s/s genotype of *5-HTTLPR* compared to HCs.

## 2. Materials and Methods

### 2.1. Participants

Patients with MDD were recruited from the Hospital of University of Occupational and Environmental Health, Japan, Kitakyushu, Japan. All patients were diagnosed using the full structured clinical interview from the Diagnostic and Statistical Manual for Mental Disorders-5 (DSM-5). Control participants were recruited using DSM-5 and none of them had any psychiatric diseases, a history of serious medical and neurological diseases, or a family history of major psychiatric or neurological diseases among their first-degree relatives. In line with the study phase protocol, no exclusion of subjects was made based on the dominant arm. The subjects in the present study overlapped with those in our published studies [13,21,29], however, no study has analyzed the correlation between amygdala subregions and blood metabolite levels.

### 2.2. Clinical Assessment and Blood Sampling

The severity of the depressive state was evaluated using the 17-item Hamilton Depression Scale (HAMD). The participants fasted and rested for at least 30 min before blood collection. We performed blood sampling between 9 a.m. and 11 a.m.

The patients’ blood was collected using a plain blood tube and plasma samples were separated by centrifugation at 2000× *g* for 20 min. Separated plasma samples were stored frozen at −80 °C in silicone-coated tubes until analysis. 

### 2.3. Genotyping

Seventy-three subjects from the neuroimaging study provided blood samples from which DNA was extracted using standard laboratory protocols. DNA was isolated from peripheral blood mononuclear cells using the QIAamp DNA Mini Kit (QIAGEN, Tokyo, Japan). Genotyping was carried out with a polymerase chain reaction (PCR), single-nucleotide polymorphism (SNP) genotyping system using the BigDye Terminator v3.1 Cycle Sequencing Kit (Life Technologies Japan, Tokyo, Japan). DNA was read using a BMG Applied Biosystems 3730xI DNA Analyzer (Life Technologies Japan, Tokyo, Japan). We used a forward primer (5′-GGC GTT GCC GCT CTG AAT GC-3′) and a reverse primer (5′-GAG GGA CTG AGC TGG ACA ACC AC-3′) for the *5-HTTLPR* polymorphism. Genotyping revealed that all the 25 patients with MDD had the s/s genotype of *5-HTTLPR*. Among the 49 HCs, 3 had l/s and 46 had s/s genotypes of *5-HTTLPR*. Therefore, 46 HCs with the s/s genotype of *5-HTTLPR* were assessed in this study.

### 2.4. MRI Acquisition

MRI data were obtained using a 3T MR system (Signa EXCITE 3T; GE Healthcare, Waukesha, WI, USA) with an 8-channel brain phased-array coil. Images were acquired using three-dimensional fast-spoiled gradient-recalled acquisition (3D-FSPGR). The acquisition parameters were: repetition time/echo time, 10/4.1 msec; flip angle, 10°; field of view, 24 cm; and resolution, 0.9 × 0.9 × 1.2 mm. All images were corrected for image distortion due to gradient nonlinearity using the “Grad Warp” software program [30].

### 2.5. Amygdala Subregion Volume

FreeSurfer ver.7.11 [31] was used to evaluate the volume of the amygdala subregion. This amygdala subregion segmentation technique, based on a prior probabilistic atlas and the Bayesian modeling approach, is fully automated [32]. The bilateral amygdala was generated in each subject for the basal, lateral, accessory basal, central, medial, cortical, and paralaminar nucleus, as well as the corticoamygdaloid transition area, anterior amygdaloid area, and the whole amygdala. The left and right substructures were separately analyzed. Furthermore, the estimated intracranial volume was also calculated using “aseg segmentation”.

### 2.6. Statistical Analysis

All descriptive statistics and statistical analyses were performed using Python ver. 3.0 [33] and EZR ver.1.54 [34]. We used the Mann–Whitney U test to compare the estimated total intracranial volume and the plasma levels of metabolites between the HC and MDD groups. Linear regression analysis was performed with amygdala volume as the objective variable, HC and MDD groups as explanatory variables, and age, sex, and estimated total intracranial volume as covariates to test for differences in amygdala volume. We also performed subgroup analyses by using linear regression analysis with amygdala volume as the objective variable, plasma metabolites as explanatory variables, and age, sex, and estimated total intracranial volume as covariates to test the relationship between blood metabolites and amygdala volume. Those with statistically significant correlations between the two groups were tested for interactions between amygdala subregion volume and plasma metabolites in the HC and MDD groups. The distribution of all the data was checked by histogram and expressed as mean (standard deviation) or median (interquartile range). We confirmed the normality of the residuals in the linear regression analysis and validated the model. For the sections where exhaustive tests on the correlation between blood substances and amygdala subregions were carried out, we used the Benjamini–Hochberg procedure [35] separately for each group and left and right side to control multiple comparisons. The test was two-tailed, and a *p*-value < 0.05 was considered statistically significant. Standardized partial regression coefficients were expressed as β.

## 3. Results

### 3.1. Demographic and Clinical Characteristics

The demographic data and clinical characteristics of the participants are presented in Table 1. There were more males than females in the MDD group. All participants in the HC group were right-handed, and only two participants in the MDD group were left-handed, indicating a bias in handedness.

### 3.2. Estimated Total Intracranial Volume and Plasma Levels of BDNF and Cortisol among Both Groups

The estimated total intracranial volume and plasma levels of BDNF and cortisol are shown in Table 2. The Mann–Whitney U test showed no significant difference in the estimated total intracranial volume and plasma levels of substance between the HC and MDD groups.

### 3.3. Amygdala Volume

**Figure 1:**
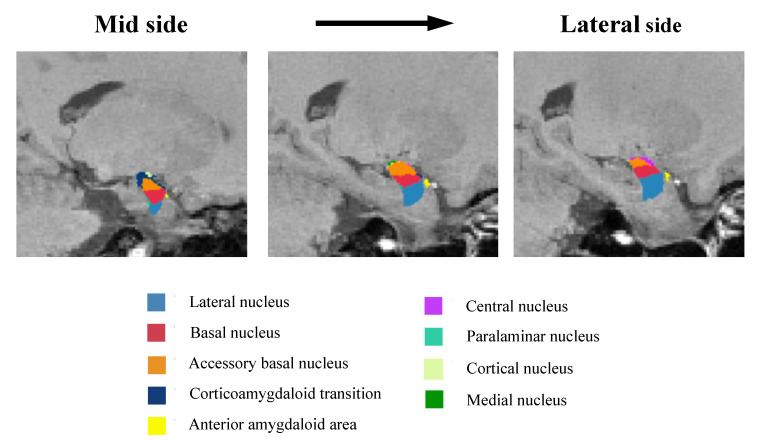
Anatomical amygdala subregions. Representative images of the amygdala subregions. The mask of each region is overlaid on sagittal T-1-Weighted images from the mid side to the lateral side.

**Figure 2:**
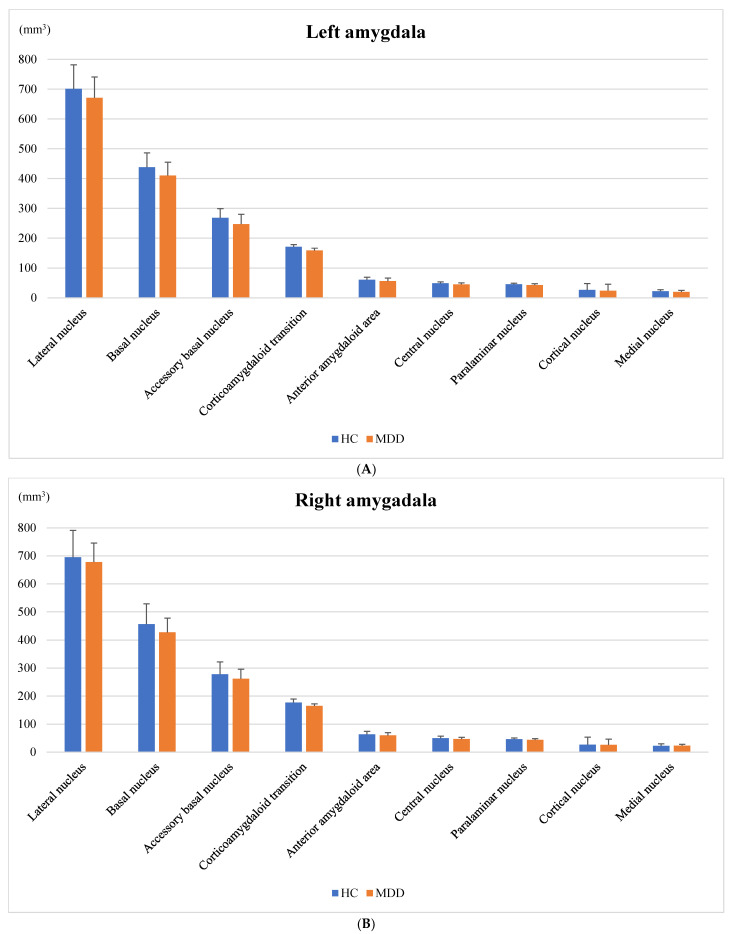
Subregion volumes in the left (**A**) and right (**B**) amygdala. (**A**) Subregion volumes in the left amygdala. Bar charts show the mean amygdala subfield volumes (mm^3^) of HCs (blue) and patients with MDD (orange). The vertical bars indicate standard deviations. Linear regression analyses showed no significant difference in all subfields between HCs and MDD patients adjusted for estimated total intracranial volume, age, and sex; (**B**) Subregion volumes in the right amygdala. Bar charts show the mean amygdala subfield volumes (mm^3^) of HCs (blue) and MDD patients (orange). The vertical bars indicate standard deviations. Linear regression analyses showed no significant difference in all subfields between HC and MDD groups adjusted for estimated total intracranial volume, age, and sex.

The anatomical amygdala subregions are shown in Figure 1. The left amygdala volume is shown in Table 3 in A (Figure 2A) and the right amygdala volume is shown in Table 3 in B (Figure 2B). There were no significant differences between the HC and MDD groups.

### 3.4. Relationship between Amygdala Volume and Plasma Levels of BDNF and Cortisol (Subgroup Analysis)

**Figure 3:**
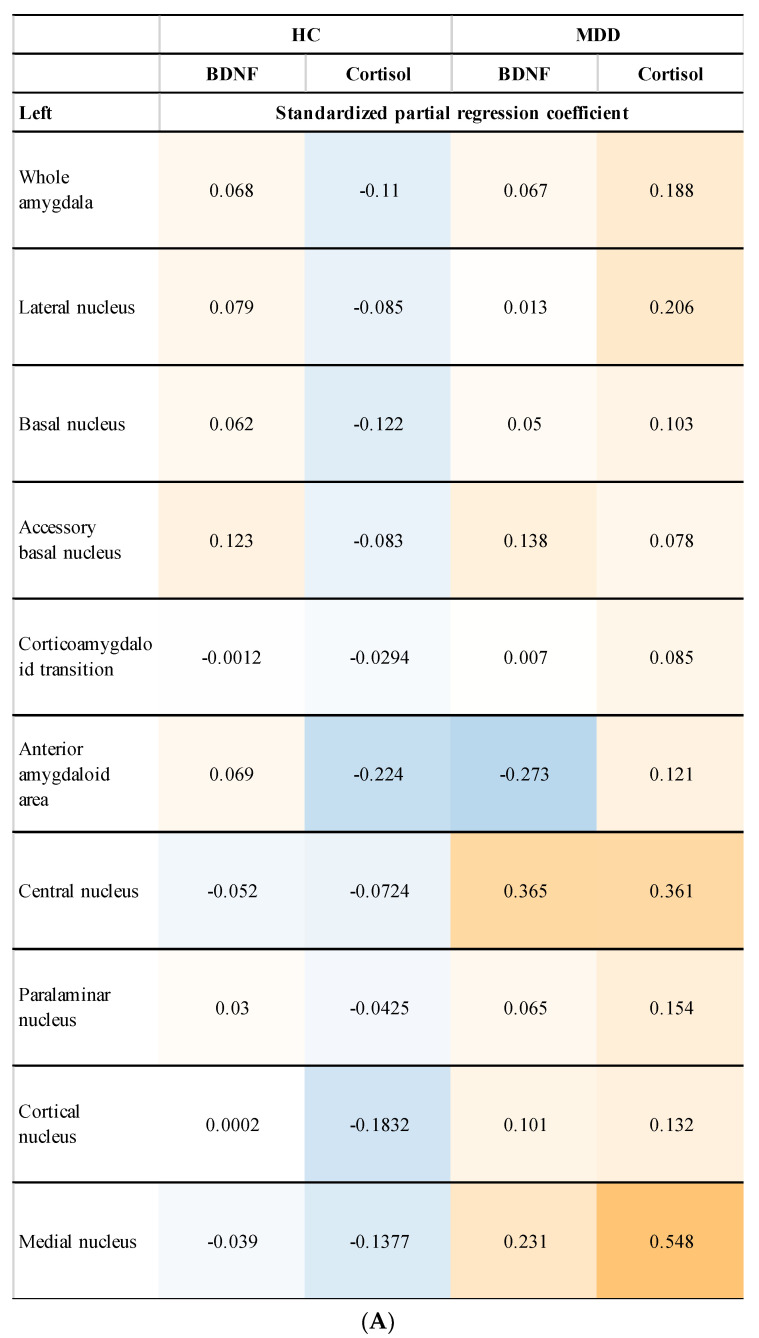

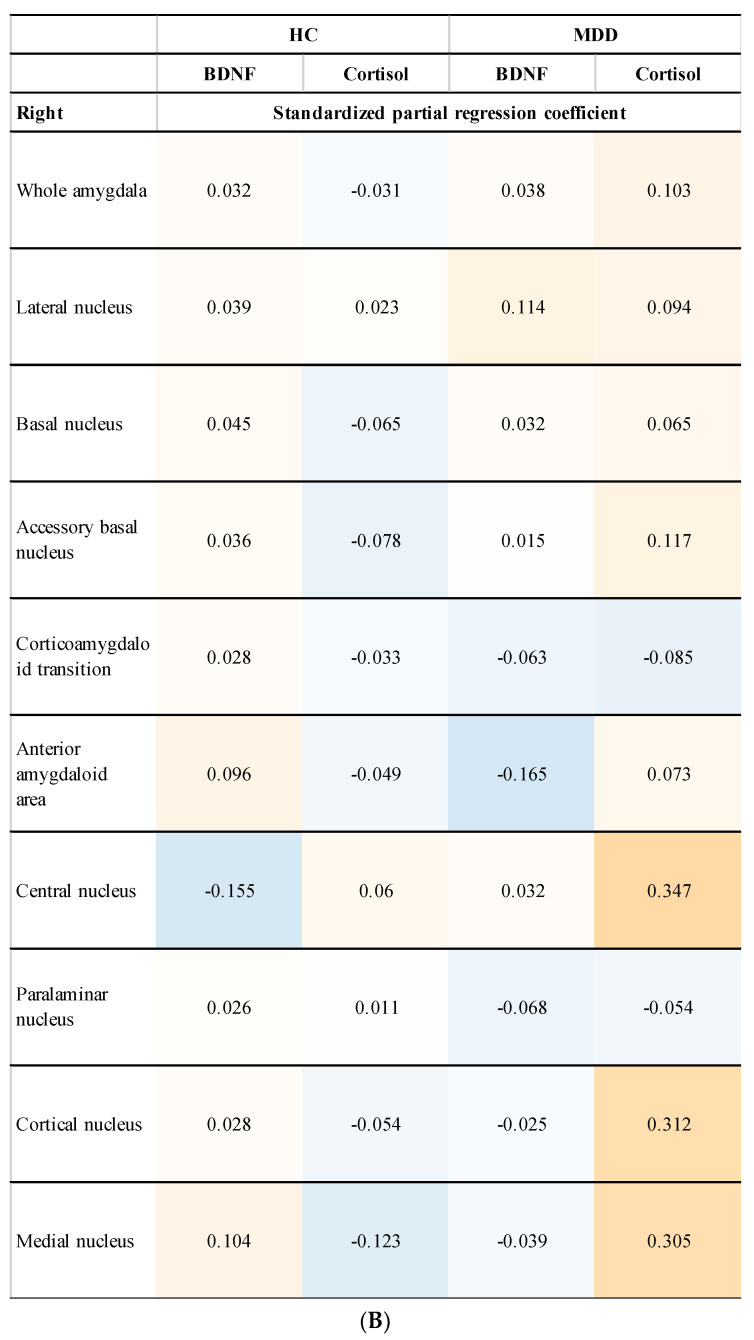
Relationship between left (**A**) and right (**B**) amygdala volume and plasma levels of BDNF and cortisol (Subgroup analysis). Plasma levels of cortisol were significantly positively correlated with the left medial nucleus (β = 0.548, *p* = 0.0082) (**A**) and right central nucleus (β = 0.347, *p* = 0.043) (**B**) in the MDD group, however, correction for multiple comparisons failed to show statistically significant differences. Positive correlations are shown in orange, negative correlations are shown in blue, and the strength of the correlation is expressed in terms of concentration (−1.0–1.0).

We showed the relationship between the plasma levels of BDNF and cortisol and the left and right amygdala volume (Figure 3). Plasma levels of cortisol were significantly positively correlated with the volume of the left medial nucleus (β = 0.548, *p* = 0.0082) and the right central nucleus (β = 0.347, *p* = 0.043) in the MDD group, however, correction for multiple comparisons with the Benjamini–Hochberg procedure did not show statistically significant differences (plasma levels of cortisol and left medial nucleus, *p* = 0.16) (plasma levels of cortisol and right central nucleus, *p* = 0.86).

## 4. Discussion

This study aimed to investigate the differences in amygdala total and subregion volumes, the relationship between amygdala total and subregion volumes in the MDD and HC groups, and the correlation between plasma levels of BDNF and cortisol and the volume of each group. The main findings are as follows: There was no statistically significant difference in amygdala total and subregion volumes between the HC and MDD groups. No correlations were found between the total and subregion amygdala volume and plasma levels of cortisol or BDNF.

The hypothalamic-pituitary-adrenal (HPA) axis has been the focus of depression research [36]. One of the most consistent biological findings in patients with severe MDD with melancholic features, with dysregulation of the HPA axis, is the increased amount of plasma cortisol. This biological difference is due to a combination of excessive stress-related cortisol secretion and impaired glucocorticoid receptor-mediated HPA axis feedback inhibition. HPA axis changes are also associated with the impaired cognitive function of MDD [37]. Growth and adaptability at a neuronal level have been more broadly termed neuroplasticity, and it is possibly this neuroplasticity at a cellular level that is altered by inflammation and HPA axis dysfunction, both caused by environmental stress [38]. The process of neurogenesis is controlled by nerve growth factors, including BDNF, which is reduced in patients with MDD [23], and reduced BDNF can be recovered with either pharmacotherapy or psychological interventions [39].

While the amygdala plays a crucial role in the pathogenesis of MDD, morphometric studies of the amygdala have yielded inconsistent results, including larger, smaller, or similar volumes in these patients compared to HCs [3]. Follow-up meta-analyses indicated that amygdala volume was significantly decreased in depression, including unmedicated and depressed participants, and significantly increased in depression when considering only studies with samples composed entirely of medicated and depressed patients [40]. Another meta-analysis showed that adults with MDD and comorbid anxiety had significantly higher amygdala volumes [41]. The volume of total and any subregions of the amygdala were not different in the MDD group that was drug-naive and experiencing a first episode compared to HC groups in the present study. It is plausible that disease progression and medications affect the volume of the amygdala. A recent study reported that the right medial nucleus volume was larger in patients with MDD than in HCs [5]. A previous study showed that connections from the amygdala to the hypothalamus activate the HPA axis and cortisol response [5]. Glucocorticoid receptors are expressed in the amygdala, similar to the hippocampus [42]. In contrast to hippocampal feedback, amygdala drive promotes hypothalamic corticotropin-releasing hormone (CRH) secretion [43].

Diminished connectivity between the amygdala and the pregenual part of the anterior cingulate cortex (pgACC) during fear-related processing could be more vulnerable to anxiety as it pertains to greater circulating cortisol levels in daily life. In short, individual functional neural connective patterns of the amygdala-hippocampal-pgACC circuit could play a role in the complicated link between cortisol and emotional-related behaviors [44]. A recent report suggested that the basolateral amygdala to subgenual anterior cingulate cortex neural connectivity and the cortisol–norepinephrine interaction, which may be associated with implicit memory bias, could be one of the pathophysiologies of anxiety disorders and MDD [45]. BDNF is known to regulate synaptic plasticity and memory formation in many areas of the brain, including the amygdala, where BDNF signaling via tyrosine kinase B receptor (TrkB) is prominently involved in fear learning [46]. BDNF, acting through the TrkB, is thought to be a critical mediator of fear learning, and amygdala TrkB activation is required for the consolidation of stable extinction memories, which is involved in the pathophysiology of psychiatric diseases, including anxiety and mood disorders [47]. Pe-synaptic TrkB in basolateral amygdala neurons has been reported to be necessary for memory extinction and contributes to BDNF signaling transduction from the basolateral amygdala to the infralimbic prefrontal cortex [48]. In fact, MDD patients showed impaired acquisition of conditioned fear [49]. The BDNF *Val66Met* polymorphism could be have an effect on the amygdala–cortical connectivity during adolescence [50]. These findings suggest that the influence of cortisol and BDNF on the amygdala could play a role in the pathophysiology of MDD. However, no significant correlations could be found in the amygdala volume and plasma cortisol of BDNF in the present explanatory research.

This study had several limitations. First, we enrolled patients with the s/s genotype of *5-HTTLPR* of first-episode and drug-naive MDD; therefore, we must perform further validation using a larger sample including the l-allele genotype of *5-HTTLPR*. Second, there was a sex ratio difference between the HC and MDD groups, and the MDD group also included two left-handed individuals. Third, we used plasma levels of BDNF and cortisol, not cerebrospinal fluid, which showed a definite discrepancy between the periphery and the brain. Finally, statistically significant differences were found in the results before multiple testing correction, so the present results could be a type 2 error due to the low detection power of the small sample sizes.

## 5. Conclusions

In summary, we aimed to investigate the differences in amygdala total and subregion volumes in Japanese patients with s/s genotype of first-episode and drug-naive MDD and a HC group. We also aimed to investigate the correlation between the volume of each group and the plasma levels of BDNF and cortisol. Our results indicate that no volume differences in total and subregion amygdala were found between the MDD group and the HC group. In addition, no correlations were found between the total and subregion amygdala volume and plasma levels of cortisol or BDNF.

## Figures and Tables

**Table 1:** Demographic data and clinical characteristics.

	HC	MDD
	(*n* = 46)	(*n* = 25)
Demographic data		
Age, years	39 (32–49.5)	42 (33–54)
Sex, male/female	34/12	14/11
Dominant hand, right/left	46/0	23/2
Smoking, smoking/non-smoking	22/24	12/13
Education, years	16 (2.8)	13 (2.5)
Clinical characteristics		
Duration of the disease, month	-	4.5 (5.6)
HAMD total score	-	22 (6.0)

All data are expressed as mean (standard deviation) or median (interquartile range). HAMD is the Hamilton Depression Scale.

**Table 2:** Estimated total intracranial volume and plasma levels of metabolites.

	HC	MDD	z-Value	*p*-Value
Estimated total intracranial volume, mm^3^	1,600,482 (146,824)	1,555,121 (136,717)	1.35	0.18
Plasma metabolites levels				
BDNF, ng/mL	4.60 (2.63–7.60)	3.55 (1.45–7.20)	1.33	0.19
Cortisol, ug/dl	8.90 (6.55–12.3)	11.8 (10.2–12.9)	−1.84	0.067

All data are expressed as median (interquartile range). BDNF is brain-derived neurotrophic factor.

**Table 3:** (**A**). Left amygdala volume (mm^3^); (**B**). Right amygdala volume (mm^3^).

**(A)**			
	**HC**	**MDD**	**Adjusted *p*-Value**
Whole amygdala	1783 (188)	1677 (172)	0.14
Lateral nucleus	701 (81)	671 (70)	0.65
Basal nucleus	438 (48)	410 (45)	0.11
Accessory basal nucleus	268 (31)	247 (33)	0.052
Corticoamygdaloid transition	171 (21.8)	159 (21.9)	0.15
Anterior amygdaloid area	61.0 (7.62)	56.3 (7.49)	0.071
Central nucleus	48.9 (8.73)	45.2 (10.1)	0.42
Paralaminar nucleus	45.5 (5.38)	43.1 (4.87)	0.38
Cortical nucleus	26.5 (3.63)	24.2 (4.64)	0.17
Medial nucleus	22.3 (5.02)	20.3 (5.31)	0.43
**(B)**			
	**HC**	**MDD**	**Adjusted *p*-value**
Whole amygdala	1813 (259)	1734 (182)	0.77
Lateral nucleus	695 (96)	678 (68)	0.64
Basal nucleus	456 (73)	427 (51)	0.43
Accessory Basal nucleus	278 (44)	262 (34)	0.59
Corticoamygdaloid transition	177 (26.9)	165 (20.4)	0.28
Anterior amygdaloid area	63.3 (12.5)	59.9 (7.22)	0.62
Central nucleus	49.6 (11.1)	46.6 (9.79)	0.27
Paralaminar nucleus	46.3 (7.53)	43.7 (5.25)	0.56
Cortical nucleus	26.7 (4.52)	26.3 (4.36)	0.47
Medial nucleus	22.4 (7.74)	23.1 (6.31)	0.41

All data are expressed as mean (standard deviation). All *p*-values were adjusted for age, sex, and the estimated total intracranial volume.

## Data Availability

The data that support the findings of this study are available from the corresponding author, upon reasonable request.

## Abbreviations

BDNF	brain-derived neurotrophic factor
CRH	corticotropin-releasing hormone
DSM-5	Diagnostic and Statistical Manual for Mental Disorders-5
HAMD	Hamilton Depression Scale
HC	healthy control
HPA	hypothalamic-pituitary-adrenal
LCSPT	limbic-cortico-striatal-pallidal-thalamic
MDD	major depressive disorder
MRI	magnetic resonance imaging
PCR	polymerase chain reaction
pgACC	part of the anterior cingulate cortex
SNP	single-nucleotide polymorphism
TrkB	tyrosine kinase B receptor
3D-FSPGR	three-dimensional fast-spoiled gradient-recalled acquisition
5-HT	serotonin
5-HTT	serotonin transporter
*5-HTTLPR*	serotonin transporter gene polymorphism
